# Multi-breed host rumen epithelium transcriptome and microbiome associations and their relationship with beef cattle feed efficiency

**DOI:** 10.1038/s41598-023-43097-8

**Published:** 2023-09-27

**Authors:** P. A. S. Fonseca, S. Lam, Y. Chen, S. M. Waters, L. L. Guan, A. Cánovas

**Affiliations:** 1 Centre for Genetic Improvement of Livestock, Department of Animal Biosciences, University of Guelph, Guelph, ON N1G 2W1 Canada; 2 Livestock Gentec, Department of Agriculture, Food & Nutritional Science, University of Alberta, Edmonton, AB T6H 2P5 Canada; 3 Teagasc, Animal and Bioscience Research Department, Animal and Grassland Research and Innovation Centre, Grange, Dunsany, C15 PW93 Co. Meath Ireland

**Keywords:** Animal breeding, Functional genomics, Microbial genetics, Sequencing, RNA sequencing

## Abstract

Understanding host-microbial interactions in the rumen and its influence on desirable production traits may lead to potential microbiota manipulation or genetic selection for improved cattle feed efficiency. This study investigated the host transcriptome and its correlation with the rumen archaea and bacteria differential abundance of two pure beef cattle breeds (Angus and Charolais) and one composite beef hybrid (Kinsella) divergent for residual feed intake (RFI; low-RFI vs. high-RFI). Using RNA-Sequencing of rumen tissue and 16S rRNA gene amplicon sequencing, differentially expressed genes (FDR ≤ 0.05, |log_2_(Fold-change) >|2) and differentially abundant (p-value < 0.05) archaea and bacteria amplicon sequence variants (ASV) were determined. Significant correlations between gene expression and ASVs (p-value < 0.05) were determine using Spearman correlation. Interesting associations with muscle contraction and the modulation of the immune system were observed for the genes correlated with bacterial ASVs. Potential functional candidate genes for feed efficiency status were identified for Angus (*CCL17*, *CCR3*, and *CXCL10*), Charolais (*KCNK9*, *GGT1* and *IL6*), and Kinsella breed (*ESR2*). The results obtained here provide more insights regarding the applicability of target host and rumen microbial traits for the selection and breeding of more feed efficient beef cattle.

## Introduction

The projected future global demands for meat and dairy production have pressured agricultural sectors to provide more efficient and sustainable food production systems, leading to opportunities and challenges for improving cattle production systems^1,2^ such as improving cattle feed efficiency. Feed efficiency is a complex trait influenced by multiple biological mechanisms mainly attributed to metabolizable energy use and energy partitioning^3^. This suggests a systematic approach to studying feed efficiency may help better understand its underlying biological mechanisms.

Approaches using high-throughput -OMICs technologies, including RNA-Sequencing (RNA-Seq), have allowed for improved understanding of host genome and transcriptome features^4,5^. Consequently, bringing insight to associations between the host transcriptome and desirable production traits to help better understand the underlying genetic architecture and biological complexity of cattle feed efficiency^6–11^.

Due to the known relationship between the metabolic capacity for ruminants to expend energy and their symbiotic rumen microbial community^12,13^, systematic approaches have studied the rumen microbiome in association with feed efficiency. This highlights the need to better understand host genetics with other major energy sinks, like the rumen, with feed efficiency. Prior studies in beef cattle have emerged, providing a better understanding of how rumen microbial features and host genetics interact^14–18^.

The host genetic influence on rumen microbial features and its association with feed efficiency in beef cattle has been recently reported, where 19 single nucleotide polymorphism (SNPs) in the host genome were associated with 14 rumen microbial taxa. Among the identified SNPs, five were co-localized with known quantitative trait loci (QTL) for cattle feed efficiency^14^. In addition, Li et al.^14^ revealed distinguishable differences in the rumen microbial features across beef cattle breeds and residual feed intake (RFI) groups. The RFI is defined the difference between the actual and the predicted feed intake required to obtain rate of gain^19^. Consequently, animals with negative RFI values are consuming less than the predicted consumption and consequently, are more feed efficient. On the other hand, animals with positive RFI consume more than the predicted consumption and are less feed efficient. Li et al.^14^ was the first study to characterize bovine genotype with heritable microbial features with feed efficiency in cattle. Some studies have also estimated the heritability of rumen microbiome features, revealing low to moderate heritabilities^14,20–22^ suggesting its ability to be genetically inherited. With evidence of the rumen microbiome being partially influenced by host genetics^14,22^ and the potential for rumen microbial traits to be heritable, it is possible that the rumen microbiome can be genetically inherited and a specific rumen microbial profile may be optimal for improved feed efficiency^22^. These findings highlight the importance to study host-microbiome genetic interactions and their influence on feed efficiency in beef and dairy cattle and highlight the potential to either modify the rumen microbiome or select for it to improve efficiency. Understanding host-microbial interactions in the rumen and its influence on desirable production traits may lead to potential microbiota manipulation or genetic selection for improved cattle feed efficiency.

The abundance of studies aiming to discover the arcane interactions underlying feed efficiency suggests that knowledge is still limited on the impacts of variations in the rumen microbiota on host biology and function. Further approaches to understanding host-microbial interactions in the rumen and its influence on desirable production traits may lead to potential microbiota manipulation or genetic selection for improved cattle feed efficiency^23^.

The objectives of this study were to: (1) Determine significantly differentially expressed (DE) genes across divergent feed efficiency groups in two pure beef cattle breeds and one composite hybrid; (2) Determine differentially abundant rumen archaeal and bacterial amplicon sequence variant (ASV) abundance between divergent feed efficiency groups in two pure beef cattle breeds and one composite hybrid; (3) Identify significant correlations between the archaea or bacteria differentially abundant ASV (rumen microbial metagenome) and gene expression (host functional transcriptome) between divergent feed efficient cattle to determine ‘host-microbiome genetic interactions’ that may be influencing feed efficiency; and 4) Perform functional annotation of the genes that were significantly associated with the differentially abundant ASVs.

## Results and discussion

### RNA-sequencing and Amplicon-sequencing alignment statistics

On average, the total number of reads mapped for all samples in low and high-RFI animals were 303,428,164 and 318,958,134, for Angus (AN); 317,776,686 and 299,963,316 for Charolais (CH); and 328,016,010 and 332,007,994 for Kinsella (KN). The majority of reads were successfully uniquely mapped to the bovine reference genome (ARS-UCD2.1 release 97) which was on average, 86.68% for low-RFI and 91.02% for high-RFI for AN, 91.22% for low-RFI and 90.22% for high-RFI for CH, and 90.64% for low-RFI and 90.65% for high-RFI for KN (Supplementary Table 1). The average number of paired-end reads per sample was 39,586,465 ± 5,152,459 and the percent of uniquely mapped reads to the bovine reference genome (ARS-UCD2.1 release 97) was 90.07 ± 5.12%. As expected, an increase in the percentage of uniquely mapped reads was observed in this study using the new bovine reference genome (ARS-UCD2.1 release 97) compared to previous studies^24,25^, due to the improved gene annotation and sequence quality of the new reference.

The alignment statistics of the Amplicon-Seq analysis for each rumen content sample is shown in Supplementary Table 2. The average number of sequenced reads, total reads mapped, and percent uniquely mapped per sample for AN was 59,784.38 ± 8763.46, 48,535.63 ± 7168.28, and 81.20%, respectively. For CH, the average number of sequenced reads, total reads mapped, and percent uniquely mapped was 63,585.00 ± 5929.53, 51,635.44 ± 4767.78, and 81.22%, respectively. Finally, for KN the average number of sequenced reads, total reads mapped, and percent uniquely mapped was 59,831.63 ± 8976.15, 48,413.31 ± 7207.97, and 80.93%, respectively. Similarly, Li et al.^15^ studied the rumen microbial metagenome and identified 54.63 ± 1.42 million sequence reads after quality control, and reported 78.47 ± 0.26% of reads mapped back to assembly contigs.

### Differentially expressed genes between high-RFI and low-RFI groups

The statistical and biological significance thresholds (FDR < 0.05, and |log_2_(FC)|> 2) resulted in the identification of DEG exclusively for the Kinsella breed. For Angus and Charolais, only the biological significance threshold was reached. The DEG identified for Kinsella breed and the top 20 genes with a |log_2_(FC)|> 2 for Charolais and Angus breeds are shown on Table 1. In total, 578 (445 downregulated and 133 upregulated), 426 (274 downregulated and 152 upregulated) and 641 (358 downregulated and 283 upregulated) genes showed an |log_2_(FC)|> 2 for Angus, Charolais and Kinsella, respectively (Supplementary Table 3).

**Table 1:** Differentially expressed genes or top 20 genes based on absolute log2(fold-change) values between the rumen tissue samples of high- and low-RFI groups for Angus, Charolais and Kinsella breeds.

Ensembl ID	Gene symbol	P-value	FDR	log2(FC)	Breed
ENSBTAG00000046725	*TNNC2*	5.61 × 10^−7^	5.62 × 10^−3^	− 5.93	Kinsella
ENSBTAG00000018204	*MYH1*	6.20 × 10^−7^	5.62 × 10^−3^	− 5.85	Kinsella
ENSBTAG00000013921	*CKM*	7.30 × 10^−7^	5.62 × 10^−3^	− 4.05	Kinsella
ENSBTAG00000021218	*MYLPF*	9.53 × 10^−7^	5.62 × 10^−3^	− 5.65	Kinsella
ENSBTAG00000018369	*MYL2*	1.72 × 10^−6^	5.62 × 10^−3^	− 5.77	Kinsella
ENSBTAG00000052709	–	1.91 × 10^−6^	5.62 × 10^−3^	− 5.70	Kinsella
ENSBTAG00000007090	*MYH2*	2.33 × 10^−6^	5.62 × 10^−3^	− 5.41	Kinsella
ENSBTAG00000010880	*TNNI2*	2.81 × 10^−6^	5.62 × 10^−3^	− 5.41	Kinsella
ENSBTAG00000009707	*MYL1*	3.00 × 10^−6^	5.62 × 10^−3^	− 5.80	Kinsella
ENSBTAG00000022158	*TNNT3*	3.06 × 10^−6^	5.62 × 10^−3^	− 4.92	Kinsella
ENSBTAG00000045757	*TNNC1*	3.08 × 10^−6^	5.62 × 10^−3^	− 5.24	Kinsella
ENSBTAG00000014547	*PGAM2*	3.15 × 10^−6^	5.62 × 10^−3^	− 5.15	Kinsella
ENSBTAG00000009703	*MYH7*	3.25 × 10^−6^	5.62 × 10^−3^	− 5.48	Kinsella
ENSBTAG00000022244	*ACTN3*	3.26 × 10^−6^	5.62 × 10^−3^	− 5.07	Kinsella
ENSBTAG00000048585	–	3.28 × 10^−6^	5.62 × 10^−3^	− 5.31	Kinsella
ENSBTAG00000006541	*ATP2A1*	3.30 × 10^−6^	5.62 × 10^−3^	− 3.96	Kinsella
ENSBTAG00000046332	*ACTA1*	3.60 × 10^−6^	5.78 × 10^−3^	− 5.05	Kinsella
ENSBTAG00000031573	*NMRK2*	8.23 × 10^−6^	1.25 × 10^−2^	− 5.21	Kinsella
ENSBTAG00000020080	*MYBPC2*	9.62 × 10^−6^	1.38 × 10^−2^	− 5.04	Kinsella
ENSBTAG00000005534	*ENO3*	1.15 × 10^−5^	1.57 × 10^−2^	− 3.70	Kinsella
ENSBTAG00000005333	*MB*	3.11 × 10^−5^	4.04 × 10^−2^	− 3.84	Kinsella
ENSBTAG00000020223	*CASQ1*	3.30 × 10^−5^	4.09 × 10^−2^	− 3.99	Kinsella
ENSBTAG00000018369	*MYL2*	1.58 × 10^−3^	1.00	− 4.22	Angus
ENSBTAG00000018204	*MYH1*	2.84 × 10^−3^	1.00	− 4.05	Angus
ENSBTAG00000046332	*ACTA1*	2.74 × 10^−3^	1.00	− 4.03	Angus
ENSBTAG00000021218	*MYLPF*	2.73 × 10^−3^	1.00	− 4.00	Angus
ENSBTAG00000045757	*TNNC1*	1.37 × 10^−3^	1.00	− 3.98	Angus
ENSBTAG00000009707	*MYL1*	1.65 × 10^−3^	1.00	− 3.97	Angus
ENSBTAG00000031573	*NMRK2*	2.62 × 10^−3^	1.00	− 3.97	Angus
ENSBTAG00000007090	*MYH2*	1.42 × 10^−3^	1.00	− 3.96	Angus
ENSBTAG00000052488	*SPINK9*	5.28 × 10^−4^	1.00	− 3.96	Angus
ENSBTAG00000052709	–	1.28 × 10^−3^	1.00	− 3.95	Angus
ENSBTAG00000011392	*MYBPC1*	4.06 × 10^−3^	1.00	− 3.95	Angus
ENSBTAG00000010880	*TNNI2*	1.78 × 10^−3^	1.00	− 3.94	Angus
ENSBTAG00000009703	*MYH7*	1.55 × 10^−3^	1.00	− 3.94	Angus
ENSBTAG00000011734	*ANKRD1*	3.45 × 10^−3^	1.00	− 3.92	Angus
ENSBTAG00000046725	*TNNC2*	2.99 × 10^−3^	1.00	− 3.92	Angus
ENSBTAG00000022158	*TNNT3*	1.20 × 10^−3^	1.00	− 3.80	Angus
ENSBTAG00000022244	*ACTN3*	1.39 × 10^−3^	1.00	− 3.80	Angus
ENSBTAG00000005333	*MB*	1.57 × 10^−3^	1.00	− 3.74	Angus
ENSBTAG00000008394	*MYL3*	1.32 × 10^−2^	1.00	− 3.72	Angus
ENSBTAG00000007782	*MYOT*	6.05 × 10^−3^	1.00	− 3.70	Angus
ENSBTAG00000037768	*MMP3*	1.93 × 10^−4^	4.82 × 10^−1^	− 4.46	Charolais
ENSBTAG00000032642	–	1.09 × 10^−3^	1.00	− 4.18	Charolais
ENSBTAG00000046633	–	1.13 × 10^−3^	1.00	− 3.87	Charolais
ENSBTAG00000021565	–	1.34 × 10^−2^	1.00	− 3.23	Charolais
ENSBTAG00000005353	*DES*	4.90 × 10^−4^	6.68 × 10^−1^	− 2.81	Charolais
ENSBTAG00000039406	–	4.56 × 10^−4^	6.61 × 10^−1^	2.69	Charolais
ENSBTAG00000015441	*ACTG2*	4.49 × 10^−4^	6.61 × 10^−1^	− 2.67	Charolais
ENSBTAG00000051421	*PCP4*	7.05 × 10^−3^	1.00	− 2.65	Charolais
ENSBTAG00000033803	*FABP7*	4.54 × 10^−3^	1.00	− 2.64	Charolais
ENSBTAG00000027279	–	1.23 × 10^−2^	1.00	− 2.61	Charolais
ENSBTAG00000006546	*GSTA2*	3.57 × 10^−3^	1.00	− 2.57	Charolais
ENSBTAG00000011207	*CNN1*	3.37 × 10^−4^	6.40 × 10^−1^	− 2.49	Charolais
ENSBTAG00000046124	*KRT36*	4.19 × 10^−5^	3.74 × 10^−1^	− 2.18	Charolais
ENSBTAG00000047121	–	7.73 × 10^−3^	1.00	− 2.13	Charolais
ENSBTAG00000015988	*MYH11*	5.49 × 10^−5^	3.74 × 10^−1^	− 2.12	Charolais
ENSBTAG00000011424	*TPM2*	7.77 × 10^−5^	3.96 × 10^−1^	− 2.11	Charolais
ENSBTAG00000044123	*HAND2*	3.79 × 10^−2^	1.00	− 2.09	Charolais
ENSBTAG00000017305	–	1.01 × 10^−1^	1.00	− 2.09	Charolais
ENSBTAG00000049878	–	1.18 × 10^−1^	1.00	− 2.03	Charolais
ENSBTAG00000011473	*MYL9*	6.20 × 10^−6^	1.69 × 10^−1^	− 2.03	Charolais

#### Downregulated genes in low-RFI group

The 22 DEG (FDR < 0.05, log_2_(FC)|> 2) identified between high and low-RFI animals for the Kinsella breed were all downregulated in the low-RFI group (Table 1). The top 3 enriched GO terms identified for the DEG in Kinsella breed were *muscle system process* (adjusted p-value = 4.28 × 10^–37^), *muscle contraction* (adjusted p-value = 1.87 × 10^–32^), and *myofibril assembly* (adjusted p-value = 5.31 × 10^–26^). This close relationship between the DEG downregulated in the low-RFI group and the regulation of muscle tissue development and contraction is highlight by the presence of genes codifying for myosin (*MYH1*, *MYLPF*, *MYL2*, *MYH2*, *MYL1*, *MYH7*, and *MYBPC2*), actin (*ACTN3* and *ACTA1*) and troponin (*TNNI2*, *TNNC2*, *TNNT3*, and *TNNC1*) related proteins. In addition, genes codifying proteins directly related with the regulation of biological processes associated with muscular activity such as *CKM*, *ENO3*, *CASQ1* and *MB* were also identified as differentially expressed between high and low-RFI groups for Kinsella breed (downregulated in the low-RFI group). The enrichment analysis of GO terms for downregulated genes in the low-RFI group indicated that similar biological processes were involved with these genes in the three breeds. The functional grouping performed through the Jaccard correlation coefficient identified that among the top 30 GO terms associated with the downregulated genes in the three breeds a strong association with muscular development was present (Supplementary Fig. 1).

Similarly, the top 3 enriched GO terms identified for the downregulated genes in the Angus low-FRI group were also *muscle system process* (adjusted p-value = 1.02 × 10^–36^), *muscle contraction* (adjusted p-value = 5.47 × 10^–35^), and *myofibril assembly* (adjusted p-value = 2.09 × 10^–28^). It is important to highlight that for Angus, there were no genes which passed the statistical (FDR < 0.05) and biological (|log_2_(FC)|> 2) significance thresholds simultaneously. However, the analysis of the genes which passed the biological significance threshold suggest a strong association of these genes with regulation of the muscular tissue development. For example, for the Angus breed all the top 20 genes with highest |log_2_(FC)| were downregulated (Table 1). Among these genes, eight were from the gene families associated with the myosin superfamily of motor proteins (*MYL2*, *MYH1*, *MYLPF*, *MYL1*, *MYH2*, *MYBPC1*, *MYH7*, and *MYL3*), four were associated with troponin gene family (*TNNC1*, *TNNC2*, *TNNT3*, and *TNNI2*), and two were associated with actinin filaments (*ACTA1* and *ACTN3*). Additionally, interesting genes such as *MYOT* and *MB* were also identified among these genes.

There were no genes differentially expressed between high and low-RFI groups for the Charolais breed based on both statistical and biological significance criteria, as well as for Angus breed. Similar to the results obtained for Kinsella and Angus, genes responsible to encode myosin (*MYH11* and *MYL9*) and actin (*ACTG2*) related proteins were identified among the top 20 genes with highest |log_2_(FC)|.

The myosin and troponin related proteins are key regulators of the biochemical efficiency of the striate muscle^26^. The expression pattern or polymorphisms in genes related with myosin proteins were associated with feed efficiency status in diverse species, such as chickens, rainbow trout and cattle^14,27–29^. Actin, myosin and troponin are crucial actors in the processes of muscle contraction^30,31^ and might influence the rumen contractions and consequently the digesta passage rate through the rumen. The passage rate of digesta through the rumen was previously associated with the microbiota content^32^ and levels of produced methane (CH_4_)^32–34^. Interestingly, the increase of feeding, which raises the digesta passage rate, is associated with the reduction of digestibility and increasing of efficiency of the microbiota community to synthetize gasses and volatile fatty acids from fermented carbon^35,36^. Additionally, associations between CH_4_ levels and cellulose digestibility, neutral detergent fiber digestibility, energy digestibility and cell wall digestion were identified^37–39^. A detailed review about the relationship between the digesta passage rate, CH_4_ emissions and feed efficiency was published by Lovendahl et al.^40^.

Additionally, an interesting association between some of downregulated genes in the low-RFI group and the GO terms *feeding behavior* (adjusted p-value = 0.09) and *regulation of appetite* (adjusted p-value = 0.12) was observed (Supplementary Fig. 1). The downregulated genes *TCF15*, *TACR1*, *HAND2*, *CARTPT*, and *NPW* were associated with *feeding behavior*. On the other hand, the *HTR4*, *SLC22A3*, and *CARTPT* genes were associated with *regulation of appetite*. Among the downregulated genes associated with feeding behavior and regulation of appetite, the most distinguish functional roles were identified for *NPW* and *CARTPT* genes. The *NPW* codifies a neuropeptide which, in rats, increases the serum levels of prolactin, corticosterone, and stimulate food and water intake^41,42^. Additionally, experimental evidence through the intracerebroventricular administration of two residuals of NPW (NPW_23_ and NPW_30_) in rats resulted in an increased body temperature, suppression of feeding and body weight gain^43^. Regarding *CARTPT*, Alpha melanocyte-stimulating hormone (α-MSH) derivates from *CARTPT* product (cocainx10- and amphetamine-regulated transcript) and proopiomelanocortin (POMC) leads to an enhanced state of food intake and appetite inhibition^44^. Additionally, in humans, polymorphisms on *CARTPT* are associated with obesity and an alteration of the leptin effect on thermogenesis and energy expenditure^45–47^.

These results suggest a lower expression of genes with potential functional role in the regulation of rumen contraction and feeding behaviour. The frequency of rumen contractions is associated with the passage of the rumen digesta through the rumen which might influence the microbiota diversity and the concentration of vitality fatty acids (REF). Regarding feed behavior, significant correlation was observed between feed duration and frequency, meal size and eating rate with RFI in beef cattle^48–51^.

#### Upregulated genes in low-RFI group

In total, 133, 152, and 283 genes were upregulated (based on |log_2_(FC)|> 2) for Angus, Charolais and Kinsella, respectively. Enriched GO terms for upregulated genes were identified exclusively for Kinsella breed. A total of 177 GO terms were enriched for the upregulated genes in the low-RFI group in the Kinsella breed (GO-BP:105, GO-MF: 22, and GO-CC: 50). The top three enriched GO terms were *modulation of chemical synaptic transmission* (adjusted p-value = 4.49 × 10^–06^), *regulation of trans-synaptic signaling* (adjusted p-value = 4.49 × 10^–06^), and *neuron cellular homeostasis* (adjusted p-value = 2.43 × 10^–04^). The functional grouping of the top 30 GO terms annotated for the upregulated genes in the low-RFI group for Kinsella breed reinforce the association of these genes with regulation of neuronal activity with the largest functional group classified as *amine neurotransmitter GABAergic glutamatergic* (Supplementary Fig. 1). The effects of GABA in the human body results in an increase in the plasmatic levels of growth hormone, acting as an anabolic agent^52^. Interestingly, the inclusion of GABA producing bacteria as a feed additive demonstrated a potential impact to improve growth performance in ruminants through the reduction of amines and increasing anti-oxidative activity^53^. In addition, the utilization of rumen-protected GABA has beneficial effects over food intake and milk production in dairy cows^54,55^. Consequently, suggesting a potential role of these genes over food intake and energy homeostasis which might directly impact the feed efficiency status.

The upregulated genes in the low-RFI group in Angus and Charolais breeds were not associated with any enriched GO terms. The largest functional groups obtained through the Jaccard correlation coefficient were *regulation gonad and morphogenesis assembly* and *meiotic acid catabolic process* for Angus and Charolais, respectively (Supplementary Fig. 1). Despite the absence of enriched terms, the GO terms annotated for these groups of genes suggest an association with regulation cellular proliferation, cell cycle control and cellular homeostasis.

### Differential archaea and bacteria ASVs between high-RFI and low-RFI groups

The abundance of archaea and bacteria ASVs for Angus, Charolais and Kinsella obtained in the current study are shown on Supplementary Table 4. Regarding the archaea species, all breed groups had a significant difference between high and low-RFI animals (Table 2). The differential abundance analysis for bacterial ASVs at the genus level between high-RFI and low-RFI animals resulted in two, five, and five genera with significant differential abundance for Angus, Charolais and Kinsella, respectively (Table 3).

**Table 2:** Differentially abundant archaea genus in the rumen digesta of high- and low-RFI groups for Angus, Charolais and Kinsella breeds.

Specie	Mean (± SD) HRFI	Mean (± SD) LRFI	t	P-value	Breed
*Methanobrevibacter ruminantium clade*	1.153 (± 0.453)	0.975 (± 0.452)	0.789	0.443	Angus
*Methanobrevibacter boviskoreani clade*	0.172 (± 0.437)	0.338 (± 0.533)	− 0.684	0.506	Angus
*Methanobrevibacter gottschalkii clade*	0.231 (± 0.304)	0.279 (± 0.218)	− 0.365	0.721	Angus
*Methanosphaera sp. ISO3-F5*	0.108 (± 0.042)	0.104 (± 0.057)	0.149	0.884	Angus
*Methanosphaera sp. ISO3-F5*	0.089 (± 0.012)	0.075 (± 0.02)	1.699	0.117	Charolais
*Methanobrevibacter ruminantium clade*	1.164 (± 0.341)	1.296 (± 0.089)	− 1.062	0.319	Charolais
*Methanobrevibacter boviskoreani clade*	0.149 (± 0.399)	0 (± 0)	1.056	0.326	Charolais
*Methanobrevibacter gottschalkii clade*	0.26 (± 0.165)	0.262 (± 0.091)	− 0.042	0.967	Charolais
*Group12 sp. ISO4-H5*	0.067 (± 0.074)	0.042 (± 0.042)	0.828	0.425	Kinsella
*Methanosphaera sp. ISO3-F5*	0.162 (± 0.043)	0.147 (± 0.046)	0.708	0.491	Kinsella
*Methanobrevibacter gottschalkii clade*	0.267 (± 0.089)	0.315 (± 0.202)	− 0.606	0.558	Kinsella
*Methanobrevibacter ruminantium clade*	1.242 (± 0.112)	1.213 (± 0.208)	0.358	0.727	Kinsella

*HRFI* high residual feed intake, *LRFI* low residual feed intake, *SD* standard deviation, *t* t-test statistic.

**Table 3:** Differentially abundant bacteria genus in the rumen digesta of high- low-RFI groups for Angus, Charolais and Kinsella breeds.

Genus	Mean (± SD) HRFI	Mean (± SD) LRFI	t	P-value	Breed
*Bacteroidales RF16 uncultured rumen bacterium*	0.026 (± 0.017)	0.079 (± 0.055)	− 2.618	0.029	Angus
*Ruminococcus 1*	0.045 (± 0.013)	0.025 (± 0.016)	2.742	0.017	Angus
*Clostridiales vadin BB60 uncultured rumen bacterium*	0.006 (± 0.011)	0.032 (± 0.02)	− 3.270	0.008	Charolais
*Clostridiales Family XIII uncultured*	0.008 (± 0.015)	0.031 (± 0.02)	− 2.637	0.021	Charolais
*Prevotellaceae UCG-001*	0.104 (± 0.025)	0.071 (± 0.029)	2.490	0.026	Charolais
*Oribacterium*	0.092 (± 0.035)	0.06 (± 0.018)	2.300	0.043	Charolais
*Erysipelotrichaceae UCG-004*	0.07 (± 0.019)	0.106 (± 0.04)	− 2.305	0.044	Charolais
*Papillibacter*	0.072 (± 0.035)	0.123 (± 0.032)	− 3.013	0.009	Kinsella
*Ruminococcus 1*	0.372 (± 0.093)	0.257 (± 0.038)	3.225	0.010	Kinsella
*Prevotellaceae UCG-001*	0.132 (± 0.043)	0.084 (± 0.022)	2.848	0.017	Kinsella
*Ruminococcus gauvreauii group*	0.121 (± 0.042)	0.078 (± 0.019)	2.583	0.028	Kinsella
*Christensenellaceae R-7 group*	0.114 (± 0.016)	0.205 (± 0.106)	− 2.406	0.045	Kinsella

*HRFI* high residual feed intake, *LRFI* low residual feed intake, *SD* standard deviation, *t* t-test statistic.

The *Bacteroidales RF16 uncultured rumen bacterium* was differentially abundant between high and low-RFI animals in the Angus breed, with higher mean values in the low-RFI group (Table 3). Previously, the *Bacteroidales RF16* group was identified as differentially abundant in the rumen of Yak animals subject to different feed types, showing higher abundance in animals fed with forage when compared to animals fed with concentrate^56^. In the same study, the *Bacteroidales RF16* group was negatively correlated with important rumen metabolites (isobutyrate, isovalerate, L-Phenylalanine, L-Leucine, Hypoxanthine, L-Glutamate, Adrenic acid and Stearic acid). Oppositely, in sheep, the *Bacteroidales RF16* group showed higher abundance in the rumen of animals fed with concentrate feed supplement when compared with animals fed without supplement^57^. In pig models, the *Bacteroidales RF16* group showed an interesting decreasing pattern of abundance in the gut of animals subjected to heat stress^58^. The phyla Bacteroidete, which includes the genus *Bacteroidales RF16* group, is associated with reduction of endotoxins in the intestine, consequently, acting in the alleviation of inflammation and insulin resistance^59–61^. Therefore, these evidences reinforce a potential role of *Bacteroidales RF16* group in the regulation of feed efficiency in Angus breed.

The genus *Prevotellaceae UCG-001* was identified as differentially abundant between high and low-RFI animals in Charolais and Kinsella breeds (Table 3). In both breeds, high-RFI animals showed higher abundance of *Prevotellaceae UCG-001* when compared with low-RFI animals. The analysis of different portions of Yak rumen epithelium identified *Prevotellaceae UCG-001* as enriched in the rumen ventral epithelium^62^. In Holstein, the feeding of essential oils to neonatal animals resulted in an increased abundance of *Prevotellaceae*^63^. Interestingly, in pigs, high feed efficient animals showed higher abundance of *Prevotellaceae* when compared with low feed efficient animals^64^, while the opposite pattern was observed in the current study. Despite these differences, the *Prevotellaceae UCG-001* seems to play an important role in the mediation of restoration of leptin related pathways in lipid metabolism disorders as a response to insulin-supplemented diets in mice^65^. Consequently, suggesting a potential role of this genus in the control of feed efficiency in cattle through the control of feeding behavior by leptin action and lipid metabolism.

Two genera from the family *Ruminococcus* were identified as differentially abundant between high and low-RFI animals for Angus and Kinsella breeds. The genus *Ruminococcus 1* was observed to be more abundant in high-RFI animals in both breeds, while the *Ruminococcus gauvreauii group* was more abundant in the high-RFI animals for the Kinsella breed (Table 3). Bacteria from the *Ruminococcus* genus are characterized as cellulolytic and acting on the production of acetate, formate and hydrogen^66^. In pigs, the relative abundance of *Ruminococcus* genus in the gut microbiome was positively associated with feed efficiency^67^. In chickens, the relative abundance of *Ruminococcus* were higher in high-RFI animals in duodenum and ileum^68^. Additionally, in crossbreed beef steers the levels of *Ruminococcus* were significantly different between animals with contrasting average daily body weight gain and average daily dry matter intake^69^.

In Charolais, two genera from the *Clostridiales* family were identified as differentially abundant between high and low-RFI animals, showing higher abundance in low-RFI animals (*Clostridiales vadin BB60 uncultured rumen bacterium* and *Clostridiales Family XIII uncultured*). In pigs, members of *Clostridiales* family were identified as more abundant in the fecal microbiota of low-RFI animals^70,71^. Additionally, an integrative analysis of expressed genes and bacterial communities in the jejunal and cecal digesta of chickens suggested that *Clostridiales* modulates the expression levels of cytokines, the tight-junction protein *OCLN* and nutrient transporters for glucose and short-chain fatty acid uptake^72^. The other two differentially abundant genus identified for Charolais breed were *Oribacterium* and *Erysipelotrichaceae UCG-004*. The *Oribacterium* genus was identified as more abundant in the high-RFI group while *Erysipelotrichaceae UCG-004* was more abundant in the low-RFI group (Table 3). In Holstein cows, a high-starch total mixed ration treatment to induce milk fat depression resulted in an increased abundance of *Oribacterium* in the ruminal fluid^73^. Additionally, the same study identified a correlation between the abundance of *Oribacterium* and the concentration of amines and amino acids, where a negative correlation between *Oribacterium* abundance and alanine levels stands out. The identification of metabolites differentially abundant between divergent feed efficient beef steers related with amino acids metabolism suggest a relevant role of the rumen amino acid concentrations in the regulation of feed efficiency^74^. The genus abundance levels of *Erysipelotrichaceae UCG-004* in the rumen fluid of Holstein cows was identified as a key predictor of mastitis in a random forest model^75^. Additionally, in pigs fed with soluble fiber during pregnancy showed a remarkable decrease in the abundance of *Erysipelotrichaceae UCG-004* in the progeny of these animals, suggesting an intergenerational effect of the diet over the microbial community^76^.

For Kinsella, other two differentially abundant genus identified between high and low-RFI animals for Kinsella breed were *Prevotellaceae UCG-001* (higher abundance in high-RFI) and *Christensenellaceae R-7* (higher abundance in low-RFI). Previously, in Angus cattle, the relative abundance of *Prevotellaceae* in the ruminal fluid was positively correlated with rumen pH and feed-to-gain ratio^77^. Interestingly, in Holstein, the abundance of *Prevotellaceae UCG-001* in the rumen was positively correlated with total milk solid^78^. In addition, also in Holstein, the inclusion of commence (a blend of active *S. cerevisiae*, *E. lactis*, *B. subtilis*, *E. faecium*, and *L. casei*, and their fermentation products) and RX3 in the diet reduced the relative abundance of *Prevotellaceae UCG-001* in the rumen^79^. In humans, the *Christensenellaceae* genus showed to be a widespread genus with a heritable pattern and associated with health^80^. In the current study, the *Christensenellaceae R-7 group* showed a higher mean in the low-RFI group. Interestingly, in pigs, the relative abundance of *Christensenellaceae* had the most significant association with feed efficiency with higher mean in the low-RFI animals as observed here^81^. Similarly, in lambs, the *Christensenellaceae* family showed higher abundance in efficient animals when compared with inefficient animals^82^. In Nellore breed, the relative abundance of *Christensenellaceae R-7 group* was significantly different between positive and negative RFI groups^83^. In addition, the relative abundance of *Christensenellaceae* were positively correlated with acetate and negatively correlated with propionate concentrations in the rumen in a multi-breed study composed by the same breeds evaluated in the current study^14^. The acetate:proprionate ratio is highly correlated with ruminal pH and methane production in cattle^84^. Considering the importance of the ruminal pH over the feed efficiency in dairy cattle^85^, the *Christensenellaceae R-7 group* is an important ASVs to be monitored in beef cattle.

### Correlation between candidate genes and ASVs for feed efficiency in beef cattle

**Figure 1:**
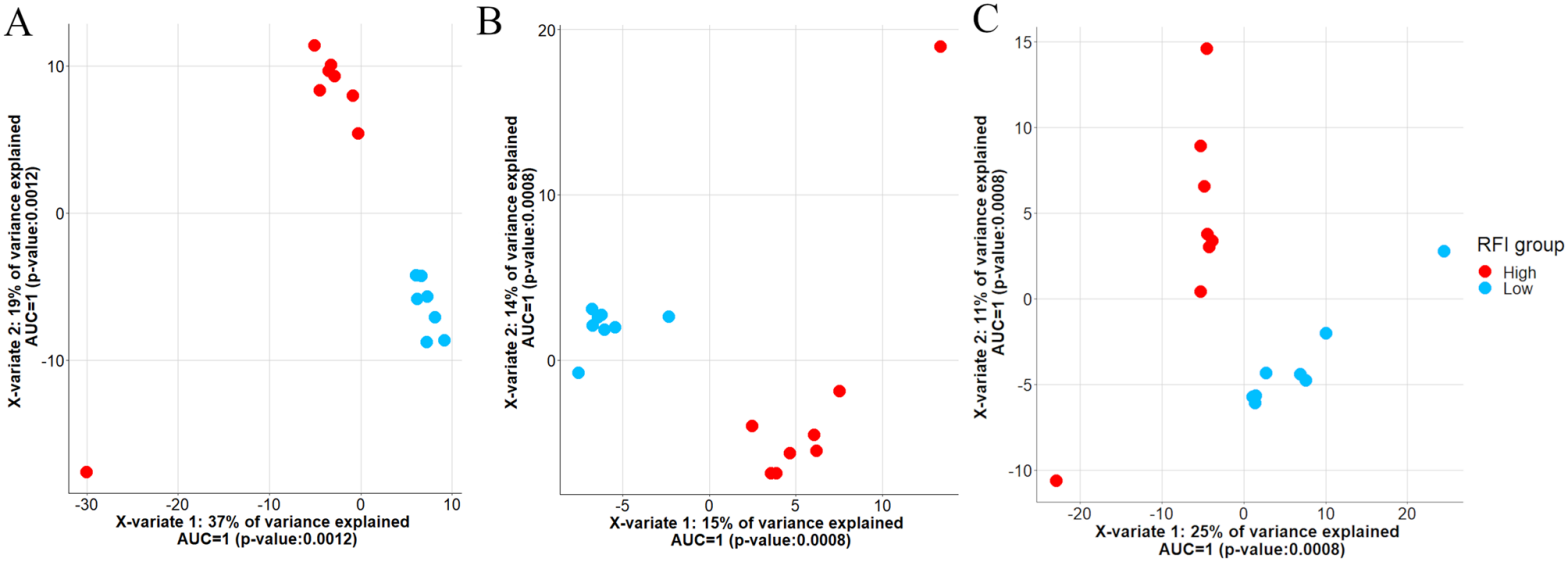
High- and low-RFI sample clustering based on gene expression levels in the rumen tissue for Angus (**A**), Charolais (**B**) and Kinsella (**C**) breeds based on the first two principal components obtained in the sPLS-DA.

The sPLS-DA analysis using the RNA-Seq counts for the genes with |log_2_(FC)|> 2 was able to perfectly discriminate high- and low-RFI animals for all the three breeds (Fig. 1). The variant selection based on the loading vectors for the first component of the discriminant analysis resulted in the selection of 462, 321, and 502 genes for Angus, Charolais and Kinsella, respectively (Supplementary Table 5). The top 10 enriched GO terms for the selected genes for each breed are shown on Table 4. The Supplementary Table 6 shows all GO terms annotated for the genes selected in the sparse partial least squares discriminant analysis (sPLS-DA). The enriched GO terms for these subsets of genes were closely related with those identified as enriched for all genes with |log_2_(FC)|> 2 and discussed previously. In summary, several GO terms associated with muscle development and regulation of muscle contraction were identified for all the breeds. As previously discussed, the regulation of muscle contraction in the rumen was associated with feed efficiency status in diverse species, such as chickens, rainbow trout and cattle^14,27–29^.

**Table 4:** Top 10 enriched gene ontology terms for the genes selected which better classify high- and low-RFI animals based on the expression in the rumen tissue in the sPLS-DA for Angus, Charolais and Kinsella breeds.

Breed	Description	GO class	P-value	FDR	Gene
Angus	Contractile fiber	CC	3.18 × 10^−53^	9.82 × 10^−51^	*MYH15, FBXO32, SCN1A, CASQ2, MYH6, KY, LMOD3, MYOZ3, TNNT3, ANKRD1, LMOD2, SYNPO2L, TRIM54, MYOZ1, MYPN, MYBPC1, RYR1, MYL2, TNNC1, MYH2, STYXL2, CMYA5, MYL3, CSRP3, MYOT, TNNI2, LRRC39, XIRP2, NRAP, TNNT1, IGFN1, ACTA1, MYLPF, ABRA, MYOM2, CAV3, TNNC2, LDB3, MYL1, ACTN3, MYOM3, MYH1, NEB, MYLK2, CASQ1, ACTN2, CACNA1S, MYOM1, KLHL41, TCAP, SMTNL1, TRIM63, CAPN3, TNNI1, MYH8, FLNC, TMOD4, MYOD1, MYOZ2*
Angus	Myofibril	CC	5.05 × 10^−53^	9.82 × 10^−51^	*MYH15, FBXO32, SCN1A, CASQ2, MYH6, KY, LMOD3, MYOZ3, TNNT3, ANKRD1, LMOD2, SYNPO2L, TRIM54, MYOZ1, MYPN, MYBPC1, RYR1, MYL2, TNNC1, MYH2, STYXL2, CMYA5, MYL3, CSRP3, MYOT, TNNI2, LRRC39, XIRP2, NRAP, TNNT1, IGFN1, ACTA1, ABRA, MYOM2, CAV3, TNNC2, LDB3, MYL1, ACTN3, MYOM3, MYH1, NEB, MYLK2, CASQ1, ACTN2, CACNA1S, MYOM1, KLHL41, TCAP, SMTNL1, TRIM63, CAPN3, TNNI1, MYH8, FLNC, TMOD4, MYOD1, MYOZ2*
Angus	Sarcomere	CC	3.81 × 10^−51^	4.94 × 10^−49^	*FBXO32, SCN1A, CASQ2, MYH6, KY, LMOD3, MYOZ3, TNNT3, ANKRD1, LMOD2, SYNPO2L, TRIM54, MYOZ1, MYPN, RYR1, MYL2, TNNC1, MYH2, STYXL2, CMYA5, MYL3, CSRP3, MYOT, TNNI2, LRRC39, XIRP2, NRAP, TNNT1, IGFN1, ACTA1, ABRA, MYOM2, CAV3, TNNC2, LDB3, MYL1, ACTN3, MYOM3, MYH1, NEB, MYLK2, CASQ1, ACTN2, CACNA1S, MYOM1, KLHL41, TCAP, SMTNL1, TRIM63, CAPN3, TNNI1, MYH8, FLNC, TMOD4, MYOZ2*
Angus	Muscle system process	BP	7.53 × 10^−37^	2.22 × 10^−33^	*STAC2, FBXO32, SLC8A3, SCN1A, SGCA, CLCN1, CASQ2, MYH6, TRIM72, LMOD3, CHRM3, CKMT2, MYBPH, TNNT3, LMOD2, SCN4B, MYOZ1, MYBPC1, RYR1, MYL2, TNNC1, MYH2, MYL3, CSRP3, MYOT, TNNI2, TNNT1, MYBPC2, ACTA1, MYLPF, MYOM2, CAV3, TNNC2, ATP2A1, MYL1, ACTN3, MYOM3, MYH1, MYLK2, CASQ1, SLN, PGAM2, ACTN2, CACNA1S, MYOM1, KLHL41, JSRP1, TCAP, GHSR, TRIM63, SCN4A, TNNI1, MYH8, TMOD4, CHRNA1, MYOD1, MLIP, MYOZ2, TACR2*
Angus	Muscle contraction	BP	3.20 × 10^−35^	4.72 × 10^−32^	*STAC2, SLC8A3, SCN1A, SGCA, CLCN1, CASQ2, MYH6, LMOD3, CHRM3, CKMT2, MYBPH, TNNT3, LMOD2, SCN4B, MYBPC1, RYR1, MYL2, TNNC1, MYH2, MYL3, CSRP3, MYOT, TNNI2, TNNT1, MYBPC2, ACTA1, MYLPF, MYOM2, CAV3, TNNC2, ATP2A1, MYL1, ACTN3, MYOM3, MYH1, MYLK2, CASQ1, PGAM2, ACTN2, CACNA1S, MYOM1, KLHL41, JSRP1, TCAP, GHSR, TRIM63, SCN4A, TNNI1, MYH8, TMOD4, CHRNA1, TACR2*
Angus	Myofibril assembly	BP	6.47 × 10^−30^	6.36 × 10^−27^	*SIX4, MYH6, LMOD3, TNNT3, ANKRD1, LMOD2, SYNPO2L, MYOZ1, MYPN, MYL2, CSRP3, NRAP, TNNT1, ACTA1, MYOM2, CAV3, LDB3, NEB, CASQ1, ACTN2, KLHL41, TCAP, CAPN3, FLNC, TMOD4, MYOZ2*
Angus	Striated muscle cell development	BP	1.04 × 10^−29^	7.69 × 10^−27^	*SIX4, MYH6, LMOD3, TNNT3, ANKRD1, LMOD2, SYNPO2L, MYOZ1, MYPN, MYL2, CSRP3, NRAP, TNNT1, ACTA1, MYOM2, CAV3, LDB3, NEB, CASQ1, ACTN2, KLHL41, TCAP, CAPN3, FLNC, TMOD4, MYOZ2*
Angus	I band	CC	1.27 × 10^−26^	1.23 × 10^−24^	*FBXO32, SCN1A, CASQ2, MYH6, KY, MYOZ3, ANKRD1, SYNPO2L, TRIM54, MYOZ1, MYPN, RYR1, MYL3, CSRP3, MYOT, XIRP2, NRAP, IGFN1, CAV3, LDB3, ACTN3, NEB, CASQ1, ACTN2, CACNA1S, TCAP, SMTNL1, TRIM63, CAPN3, FLNC, MYOZ2*
Angus	Muscle cell development	BP	2.77 × 10^−25^	1.63 × 10^−22^	*NFATC2, SIX4, MYH6, SYPL2, LMOD3, TNNT3, ANKRD1, LMOD2, SYNPO2L, MYF6, ALPK2, MYOZ1, MYPN, RYR1, MYL2, CSRP3, NRAP, TNNT1, ACTA1, MYOM2, CAV3, LDB3, ACTN3, NEB, CASQ1, ACTN2, KLHL41, TCAP, CAPN3, FLNC, TMOD4, MYOD1, MYOZ2*
Angus	Sarcomere organization	BP	4.48 × 10^−25^	2.20 × 10^−22^	*SIX4, MYH6, TNNT3, ANKRD1, LMOD2, SYNPO2L, MYOZ1, MYPN, CSRP3, TNNT1, MYOM2, CAV3, LDB3, CASQ1, ACTN2, KLHL41, TCAP, CAPN3, FLNC, MYOZ2*
Charolais	Voltagx10-gated potassium channel activity	MF	5.29 × 10^−05^	0.022	*KCNK9, KCNH3, HCN3, LRRC55, KCNA2, KCNB1, KCNJ13*
Charolais	Voltagx10-gated cation channel activity	MF	1.56 × 10^−04^	0.022	*KCNK9, KCNH3, HCN3, LRRC55, KCNA2, CACNG1, KCNB1, KCNJ13*
Charolais	Voltagx10-gated ion channel activity	MF	3.10 × 10^−04^	0.022	*KCNK9, KCNH3, HCN3, LRRC55, SCN4A, KCNA2, CACNG1, KCNB1, KCNJ13*
Charolais	Voltagx10-gated channel activity	MF	3.10 × 10^−04^	0.022	*KCNK9, KCNH3, HCN3, LRRC55, SCN4A, KCNA2, CACNG1, KCNB1, KCNJ13*
Charolais	Potassium channel activity	MF	3.16 × 10^−04^	0.022	*KCNK9, KCNH3, HCN3, LRRC55, KCNA2, KCNB1, KCNJ13*
Charolais	Tachykinin receptor activity	MF	3.35 × 10^−04^	0.022	*TACR1, TACR2*
Charolais	Cation channel activity	MF	3.39 × 10^−04^	0.022	*KCNK9, KCNH3, HCN3, LRRC55, SCN4A, MCOLN3, KCNA2, CACNG1, KCNB1, KCNJ13, PKD1L2, NALF1*
Charolais	Receptor ligand activity	MF	7.78 × 10^−04^	0.044	*IL6, EPGN, IL17B, SPP1, F2, CCL27, FGF5, IL37, XCL1, SST, TAFA4, CARTPT, LTB, IL27*
Charolais	Signaling receptor activator activity	MF	8.92 × 10^−04^	0.045	*IL6, EPGN, IL17B, SPP1, F2, CCL27, FGF5, IL37, XCL1, SST, TAFA4, CARTPT, LTB, IL27*
Charolais	Gated channel activity	MF	0.001	0.049	*KCNK9, KCNH3, HCN3, LRRC55, SCN4A, MCOLN3, KCNA2, CACNG1, KCNB1, KCNJ13, NALF1*
Kinsella	Contractile fiber	CC	1.82 × 10^−38^	7.88 × 10^−36^	*MYH4, MYPN, MYL6B, KY, MYOZ2, CAPN3, LMOD3, IGFN1, TNNT1, TNNI1, CACNA1S, TNNC1, CAV3, TNNC2, CSRP3, MYLPF, MYL2, TMOD4, LRRC39, MYL1, NEB, MYH1, MYH7, MYOM2, ACTN3, MYOT, TNNT3, TNNI2, MYL3, MYOZ1, SMTNL1, MYBPC1, ACTA1, MYH2, NRAP, RYR1, CASQ1, LMOD2, TRIM54, XIRP2, MYH8, LDB3, ANKRD1, CAVIN4, MYLK2, SYNPO2L, CMYA5, CASQ2, MYH6*
Kinsella	Sarcomere	CC	1.56 × 10^−37^	3.39 × 10^−35^	*MYH4, MYPN, KY, MYOZ2, CAPN3, LMOD3, IGFN1, TNNT1, TNNI1, CACNA1S, TNNC1, CAV3, TNNC2, CSRP3, MYL2, TMOD4, LRRC39, MYL1, NEB, MYH1, MYH7, MYOM2, ACTN3, MYOT, TNNT3, TNNI2, MYL3, MYOZ1, SMTNL1, ACTA1, MYH2, NRAP, RYR1, CASQ1, LMOD2, TRIM54, XIRP2, MYH8, LDB3, ANKRD1, CAVIN4, MYLK2, SYNPO2L, CMYA5, CASQ2, MYH6*
Kinsella	Myofibril	CC	6.20 × 10^−37^	8.95 × 10^−35^	*MYH4, MYPN, KY, MYOZ2, CAPN3, LMOD3, IGFN1, TNNT1, TNNI1, CACNA1S, TNNC1, CAV3, TNNC2, CSRP3, MYL2, TMOD4, LRRC39, MYL1, NEB, MYH1, MYH7, MYOM2, ACTN3, MYOT, TNNT3, TNNI2, MYL3, MYOZ1, SMTNL1, MYBPC1, ACTA1, MYH2, NRAP, RYR1, CASQ1, LMOD2, TRIM54, XIRP2, MYH8, LDB3, ANKRD1, CAVIN4, MYLK2, SYNPO2L, CMYA5, CASQ2, MYH6*
Kinsella	Muscle system process	BP	2.94 × ^−29^	1.04 × 10^−25^	*STAC2, MYH4, CHRM2, SSTR2, PRKAG3, MYL6B, MLIP, STAC3, P2RX2, MYOZ2, LMOD3, TNNT1, TNNI1, CACNA1S, SCN4A, TRIM72, SLN, TNNC1, CAV3, ATP2A1, TNNC2, CSRP3, MYLPF, MYL2, MYBPC2, TMOD4, MYL1, MYBPH, MYH1, MYH7, MYOM2, ACTN3, MYOT, TNNT3, TNNI2, MYL3, MYOZ1, MYBPC1, ACTA1, MYH2, CHRNA1, RYR1, CASQ1, LMOD2, MYH8, CLCN1, JSRP1, MYLK2, CKMT2, CASQ2, MYH6, SCN4B, MSTN*
Kinsella	Muscle contraction	BP	1.60 × 10^−27^	2.86 × 10^−24^	*STAC2, MYH4, CHRM2, SSTR2, MYL6B, STAC3, P2RX2, LMOD3, TNNT1, TNNI1, CACNA1S, SCN4A, TNNC1, CAV3, ATP2A1, TNNC2, CSRP3, MYLPF, MYL2, MYBPC2, TMOD4, MYL1, MYBPH, MYH1, MYH7, MYOM2, ACTN3, MYOT, TNNT3, TNNI2, MYL3, MYBPC1, ACTA1, MYH2, CHRNA1, RYR1, CASQ1, LMOD2, MYH8, CLCN1, JSRP1, MYLK2, CKMT2, CASQ2, MYH6, SCN4B*
Kinsella	Myofibril assembly	BP	1.75 × 10^−21^	2.07 × 10^−18^	*MYPN, MYOZ2, CAPN3, LMOD3, TNNT1, CAV3, CSRP3, MYL2, TMOD4, NEB, MYOM2, TNNT3, MYOZ1, ACTA1, NRAP, CASQ1, LMOD2, LDB3, ANKRD1, SYNPO2L, MYH6*
Kinsella	Striated muscle cell development	BP	2.51 × 10^−21^	2.23 × 10^−18^	*MYPN, MYOZ2, CAPN3, LMOD3, TNNT1, CAV3, CSRP3, MYL2, TMOD4, NEB, MYOM2, TNNT3, MYOZ1, ACTA1, NRAP, CASQ1, LMOD2, LDB3, ANKRD1, SYNPO2L, MYH6*
Kinsella	I band	CC	1.08 × 10^−19^	1.17 × 10^−17^	*MYPN, KY, MYOZ2, CAPN3, IGFN1, CACNA1S, CAV3, CSRP3, NEB, MYH7, ACTN3, MYOT, MYL3, MYOZ1, SMTNL1, NRAP, RYR1, CASQ1, TRIM54, XIRP2, LDB3, ANKRD1, CAVIN4, SYNPO2L, CASQ2, MYH6*
Kinsella	Skeletal muscle contraction	BP	1.64 × 10^−19^	1.17 × 10^−16^	*STAC2, STAC3, TNNT1, TNNI1, SCN4A, TNNC1, CAV3, ATP2A1, TNNC2, MYH7, ACTN3, TNNT3, TNNI2, CHRNA1, CASQ1, MYH8, JSRP1*
Kinsella	Muscle cell development	BP	7.61 × 10^−19^	4.52 × 10^−16^	*SHOX2, TBX18, MYPN, STAC3, ALPK2, P2RX2, MYOZ2, CAPN3, LMOD3, TNNT1, CAV3, CSRP3, MYL2, TMOD4, NEB, MYOM2, ACTN3, TNNT3, MYOZ1, ACTA1, NRAP, RYR1, CASQ1, LMOD2, LDB3, ANKRD1, SYNPO2L, MYH6*

Several significant correlations were observed between the arcsine square root-transformed relative abundance of the differential abundant bacterial genus and the read counts for the genes with |log_2_(FC)|> 2 for the three breeds (Supplementary Table 7). The top 10 enriched terms for each pair of correlated bacterial genus and genes are shown in Supplementary Fig. 2. In addition of GO enriched terms associated with muscle contraction as identified before, an interesting enrichment pattern for immune system related terms was identified for the genes correlated with several bacterial genus. In Angus, the genes correlated with *Ruminococcus 1* were associated with enriched GO terms such as neutrophil, granulocyte and leukocyte migration. For Charolais, enriched GO terms such as hepatic immune response (*Erysipelotrichaceae UCG-004 and Clostridiales Family XIII uncultured*), regulation of inflammatory response (*Oribacterium*), immature B cell differentiation (*Prevotellaceae UCG-001*) and myeloid leukocyte mediated immunity (*Clostridiales vadin BB60 uncultured rumen bacterium*) were identified. For Kinsella, immunity-related enriched GO terms were identified for the genes correlated with *Christensenellaceae R7 group* (neutrophil-mediated killing of gram-positive bacterium and neutrophil-mediated killing of bacterium). The important role of the immune system in the control of animal growth and efficiency have been highlighted in several species with a crucial role of the microbiome in the shaping of the immune system^86–89^. Therefore, the latter results might pinpoint potential candidate genes that contribute to the regulation of immune system components associated with the control of ruminal bacterial communities differentially abundant between divergent feed efficient animals.

**Figure 2:**
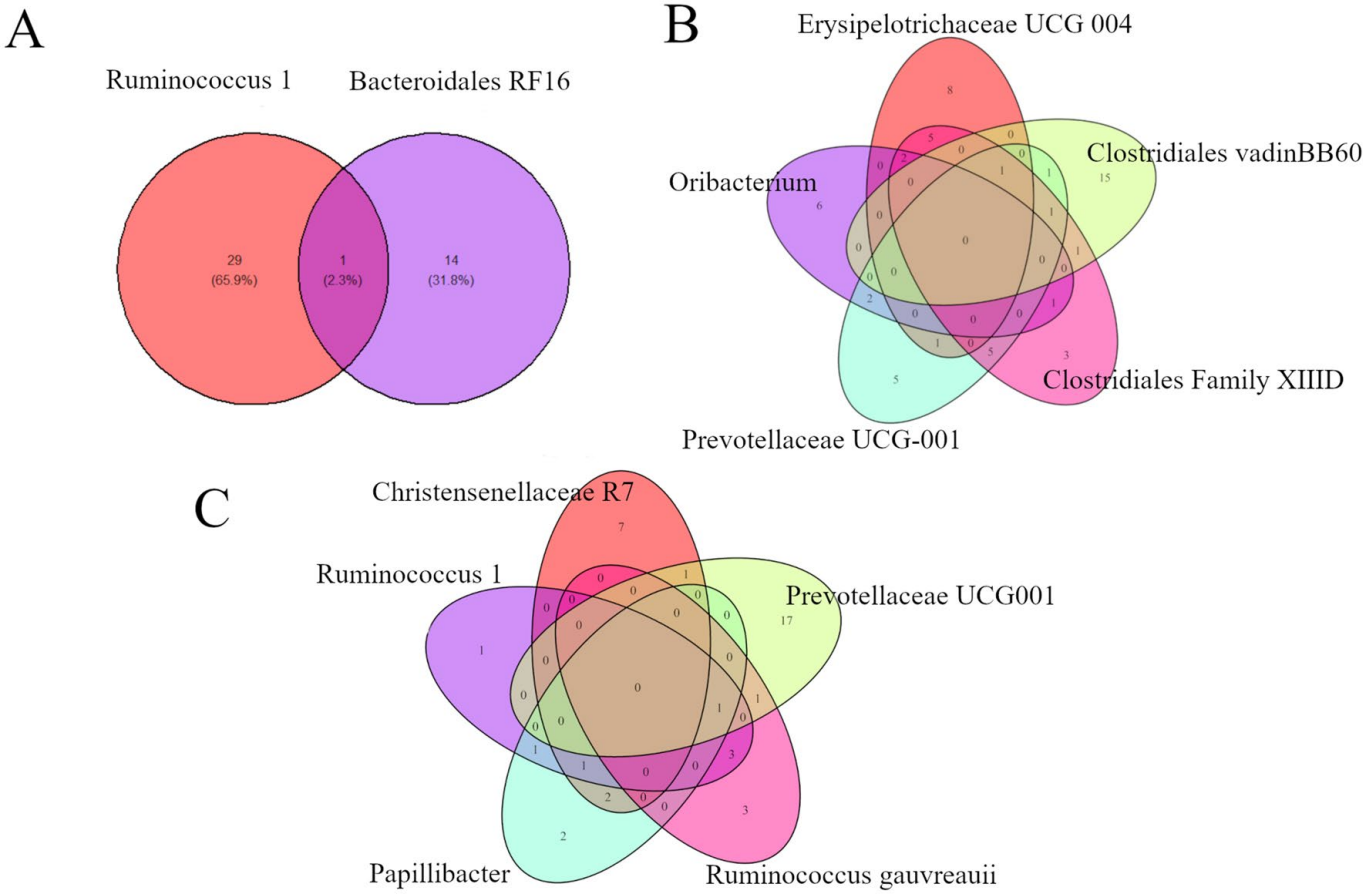
Venn diagrams showing the number of correlated genes shared between different bacterial genus for Angus (**A**), Charolais (**B**) and Kinsella (**C**) breeds.

**Figure 3:**
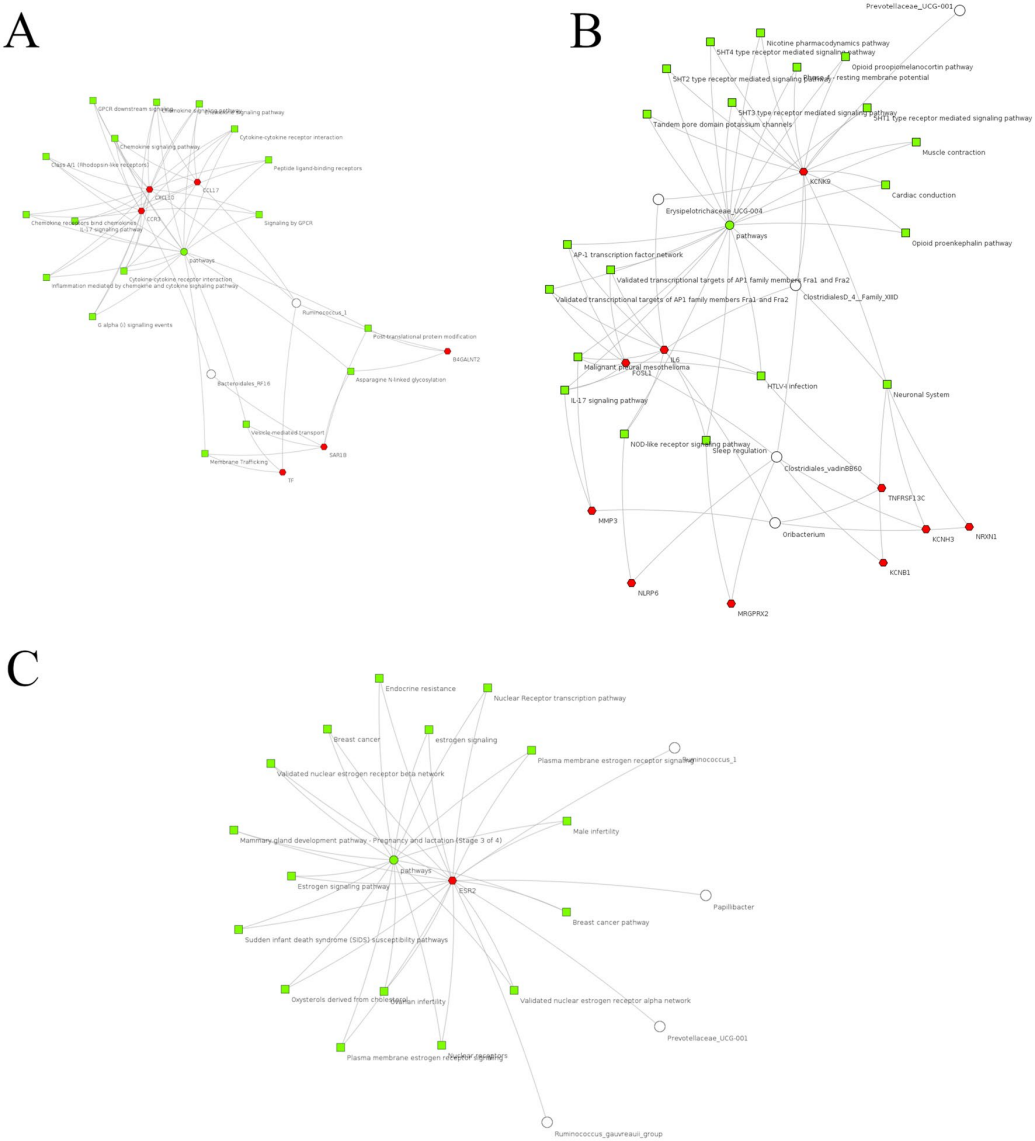
Comparative clustering of metabolic pathways (green squares) annotated for the genes (red hexagons) significantly correlated with differentially abundant ASVs (white circles) between high- and low-RFI animals for Angus (**A**), Charolais (**B**) and Kinsella (**C**).

Interestingly, there were no genes correlated with bacterial genus shared among the three breeds (all of the genes correlated with all of the bacterial genus were different among breeds). However, within breeds, common genes were identified as significantly correlated with two or more bacterial genus (Fig. 2). In addition, the comparative clustering of metabolic pathways among the genes correlated with ASVs within each breed identified relevant biological functions shared across these genes (Fig. 3). It is interesting to highlight that to build the clustering, only metabolic pathways shared between the two differentially abundant genus identified in Angus breed were included. On the other hand, for Charolais and Kinsella, only pathways shared with at least four of the five differentially abundant genus identified for each breed were included in the clustering. For Angus breed, the gene *ENSBTAG00000054941* (SCAN domain-containing protein 1-like) was simultaneously correlated with *Ruminococcus 1* and *Bacteroidales RF16 uncultured rumen bacterium*. However, the most interesting relationship between these two genera was identified when the common metabolic pathways among the correlated genes were analyzed (Fig. 3). Despite the low number of overlapping correlated genes, the two genera are chemokine and cytokine related pathways due to the correlations observed between *Ruminococcus 1* and *CCL17* (ρ = 0.62, p-value = 0.013) and *Bacteroidales RF16 uncultured rumen bacterium* with *CCR3* (ρ = 0.57, p-value = 0.30) and *CXCL10* (ρ = 0.60, p-value = 0.18). Interestingly, different cytokines and chemokines were already identified as less expressed in the rumen of beef cattle with greater average daily gain (ADG) when compared with animals with lesser ADG^90^.

For Charolais, the *KCNK9* gene was correlated with four out five differential abundant bacterial genera (*Clostridiales vadin BB60 uncultured rumen bacterium*, *Clostridiales Family XIII uncultured*, *Prevotellaceae UCG-001*, and *Erysipelotrichaceae UCG-004*). For Clostridiales *vadin BB60 uncultured rumen bacterium* (ρ = − 0.546, p-value = 0.02), *Clostridiales Family XIII uncultured* (ρ = − 0.552, p-value = 0.02), and *Erysipelotrichaceae UCG-004* (ρ = − 0.517, p-value = 0.04) positive correlations were observed. On the other hand, for *Prevotellaceae UCG-001* a negative correlation was observed (ρ = 0.571, p-value = 0.02). The *KCNK9* encodes a pH-dependent potassium channel called TASK3, which has a ubiquitous expression, but a remarkable function on T lymphocytes is observed due to its effects on downstream functions such as secretion of proinflammatory cytokines^91^. Consequently, playing an important role of the modulation of the immune system. Additionally, the genes *GGT1* and *IL6* were significantly correlated with *Clostridiales Family XIII uncultured*, *Erysipelotrichaceae UCG-004,* and *Oribacterium*. An interesting opposite correlation pattern between these genes was observed for the three genera. In *Erysipelotrichaceae UCG-004*, *GGT1* was positively correlated with the relative abundance of this genus (ρ = 0.731, p-value = 0.001), while *IL6* was negatively correlated (ρ = − 0.548, p-value = 0.027). Similarly, for *Clostridiales Family XIII uncultured GGT1* (ρ = 0.776, p-value = 0.0004) and *IL6* (ρ = − 0.601, p-value = 0.013), were also positively and negatively correlated with the relative abundance of this genus, respectively. On the other hand, for *Oribacterium*, a reversed pattern is observed, with *GGT1* (ρ = − 0.567, p-value = 0.021) being negatively correlated and *IL6* (ρ = 0.562, p-value = 0.023) being positively correlated. The *IL6* (interleukin 6) is an important inflammatory factor and was previously observed to show a significant increase in the rumen of goats and cows presenting subacute rumen acidosis^92,93^. Additionally, the metabolic pathways associated with *IL6* seems to connect the differential abundant genus identified for Charolais breed and their respective correlated genes in a single network (Fig. 3). The *GGT1* (γ-glutamyl transferase) codifies a cell surface enzyme responsible to cleave extracellular glutathione, GSH S-conjugates, among others γ-glutamyl compounds^94^. The glutathione is an antioxidant which plays crucial roles in the maintenance and regulation of thiol-redox status in the cell and contributes to the reduction of oxidative stress in dairy cows^95,96^. Despite this interesting correlation pattern, any direct relationship between *GGT1* and *IL6* was found in the literature.

In the Kinsella composite hybrid breed, the gene *ESR2* was positively correlated with three genera, which were *Ruminococcus 1* (ρ = 0.750, p-value = 0.0008), *Prevotellaceae UCG-001* (ρ = 0.627, p-value = 0.009), and *Ruminococcus gauvreauii group* (ρ = 0.661, p-value = 0.005). In addition, a negative correlation was observed between *ESR2* and *Papillibacter* (ρ = − 0.620, p-value = 0.010). Interestingly, a pivotal role of *ESR2* was observed in the connection of these differentially abundant genus through different metabolic pathways (Fig. 3). The *ESR2* gene is responsible to codify the estrogen receptor beta, a transcription factor from the steroid hormone receptor super-family. Among other function, such as reproductive-related functions, *ESR2* plays crucial roles in the glucose transporter *GLUT4* regulation and polymorphisms in this gene are associated with lipid level, insulin resistance and body fat in humans^97,98^. Additionally, the expression of *ESR2* was identified as inhibited in the rumen papilla of weaned cows, when compared with suckling calves^99^. In mice, the knockout of *ESR1*, a paralogous of *ESR2*, resulted in different levels of bacteria in the seminal fluid and gut^100^. Taken together, these results may suggest a potential role of *ESR2* in the regulation of bacterial communities in the rumen and a possible association with the control of feed efficiency status in beef cattle.

The results discussed here were obtained through a multi-breed comparison. Despite all the animals being raised under the same environmental and management conditions it is not possible to exclude the existence of hidden variables which might affect the rumen gene expression and microbiome diversity. These differences might be responsible, at least in part, by the relatively low overlapping of the results between breeds. However, these results also suggest a potential strong breed dependent effect that must be in deep investigated in further studies. Additionally, it is important to highlight that we are investigating the expression profile at rumen epithelium and generalizations regarding the direction and magnitude of the functional effect of the candidate genes in the whole organism scale must be carefully performed. The rumen epithelium is an important part of the components affecting the nutritional status^48^ and both ASVs and DEG identified here has the potential to directly act over biological mechanisms responsible to regulate feed efficiency in beef cattle.

## Conclusion

Studies on the genetic architecture of the rumen epithelium microbiome and host interactions and how these interactions influence production traits, such as feed efficiency, are newly emerging. Integration of metagenomics and host-transcriptome can provide the taxonomic and functional profile of important components of the complex system responsible for the differences observed between high and low efficient animals. At the transcriptome level, despite Angus and Charolais don’t reach the statistical threshold (FDR < 0.05) to identify DEG the analysis of genes with a significant biological threshold (|log_2_(FC)|> 2) suggested similar processes differentially expressed between HRFI and LRFI groups for all the three breeds. The regulation of rumen contraction and feed behaviour were associated with the abovementioned genes, mainly through downregulation in the low-RFI group, in all the three cattle. The metagenomic analysis indicated that only bacterial ASVs (at genus level) were differentially abundant between the feed efficiency groups in all the three breeds in the rumen epithelium. Therefore, the current study, using a multi-breed approach, identified significantly correlated host genes associated with RFI with differentially abundant rumen bacteria ASVs at genus level. The functional profile suggest that these genes may be interacting with the host microbiome, modulating the feed efficiency status across beef cattle breeds. Overall, the results of this study help to better understand the biological processes associated with the microbial dynamics in the rumen epithelium and the production response in the host. Additionally, it may suggest the application of rumen microbiome features that lead to desirable host gene function and phenotypes, providing more insight on targeting both host and rumen epithelium microbial traits that lead to the selection and breeding of more feed efficient beef cattle. Further studies on host-microbiome interactions and complimentary systems biology analysis can lead to a specific host and rumen microbiome profile which is characterized for high feed efficient cattle. Additionally, the characterization of breed potential dependent effects over the microbiome and host interaction in the rumen epithelium should be further investigate to provide better management strategies to improve the feed efficiency in each breed.

## Materials and methods

### Animal, sampling, and RFI measurement information

The animal and sampling information, as well as the Ethics Committee approval information, has been described in detail by Sun et al.^25^. All animals used in this study were managed based on the guidelines established by the Canadian Council of Animal Care and the experimental procedures were approved by the University of Alberta Livestock Animal Care and Use Committee (Protocol No.: AUP00000927). The experimental design and the analyses performed in the current study are in accordance with ARRIVE guidelines. Briefly, a total of 48 beef steers from a herd of 738 cattle cohort from the Roy Berg Kinsella Research Ranch, University of Alberta (Alberta, Canada) were used in this study. The beef steer breeds included two pure beef cattle breeds; Angus (AN; n = 16), and Charolais (CH; n = 16), and one composite hybrid Kinsella (KN; n = 16). Within each breed, from the 16 total animals, 8 animals were classified in the high RFI group and 8 in the low RFI group. The Kinsella herd is a composite hybrid, consisting of Angus, Charolais, Galloway, Hereford, Holstein, Brown Swiss, and Simmental breeds^3^.

The RFI estimates were calculated using data recorded for dry matter intake (DMI), which were collected using an automated feeding system (GrowSafe system Ltd. Airdrie, Alberta, Canada), through a 70-d period using the RFI model described by Lancaster et al.^101^. Rumen epithelium samples were collected from all animals within 30 min of slaughter after the experiment, which were immediately submerged in liquid nitrogen and then stored at -80°C until RNA isolation.

### RNA extraction, library construction, and sequencing of rumen epithelium RNA-Seq data

Detailed information on RNA extraction, library construction and sequencing are described by Sun et al.^25^. Briefly, 100 mg of rumen epithelium tissue was ground to fine powder using sterilized mortar, and total RNA was isolated using mirVana total RNA Isolation Kit (Ambion, Carlsbad, CA, USA) following the manufacturer’s instructions. The RNA was quantified using the Qubit 2.0 Fluorometer (Invitrogen, Carlsbad, CA, USA) and checked for purity and integrity using the Agilent 2200 TapeStation (Agilent Technologies, Santa Clara, CA, USA). The RNA with the ratio of 28S/18S rRNA ratio ranging from 1.7 to 2.4 and the RNA integrity number > 7.0 was used for RNA-Seq library construction. One microgram of isolated total RNA per sample was used to construct a cDNA library for RNA-Seq according to the protocol of TruSeq Stranded Total RNA Sample Prep Kit (Illumina, San Diego, CA, USA). The cDNA libraries with qualified concentration (≥ 2 nM) were performed in 16 lanes (12 samples/lane) on Illumina HiSeq 4000 sequencing platform (Illumina, San Diego, CA, USA) to obtain paired-end reads (2 × 100 bp, average Phred quality score ≥ 33) at the McGill University and Genome Quebec Innovation Centre (Montreal, Quebec, Canada).

### Reads mapping, assembling and annotation of rumen epithelium RNA-Seq data

The mRNA sequence reads were aligned to the new bovine reference genome (*Bos Taurus* ARS-UCD 1.2. bovine reference genome) with the ENSEMBL annotation tool (http://www.ensembl.org/info/data/ftp/index.html) using CLC Genomics Workbench (CLC Version 12.0.2., Aarhus, Denmark). Quality control analysis was performed in all samples as described by Cánovas et al.^102^. Briefly, quality control was performed using the ‘NGS quality control’ tool of CLC Genomics Workbench (CLC Version 12.0.2., Aarhus, Denmark), which assessed GC content (50% GC base content and less than 0.1% over-represented sequences), ambiguous base content, same length reads (100 bp), Phred score (Phred ≥ 33), base coverage (100% coverage in all bases), nucleotide contributions and over-represented sequence parameters (25% of A, T, G and C nucleotide contributions)^102^. One sample from the low-RFI Angus group (ID = 407) was removed from the study due to poor alignment quality (uniquely mapped reads < 80%). The remaining samples (n = 47) passed the quality control analysis according to the conditions stated above^5^.

### Differential gene expression analysis of rumen epithelium RNA-Seq data for low and high-RFI gene modules

Using the ‘Empirical Analysis of differential Gene Expression’ tool in CLC Genomics Workbench (CLC Version 12.0.2., Aarhus, Denmark), differentially expressed genes (DEG) were identified between low-RFI (more feed efficient) and high-RFI (less feed efficient) animals for each breed (AN low-RFI n = 7; high-RFI n = 8; CH low-RFI n = 8; high-RFI n = 8; KN low-RFI n = 8; high-RFI n = 8) by t-test. Gene expression levels were quantified in reads per kilo base per million mapped reads (RPKM). Genes were considered significantly differentially expressed between low and high-RFI groups when they met the following statistical and biological significance thresholds: False Discovery Rate (FDR) q < 0.05, and log_2_ of fold-change (|log_2_(FC)|) > 2.

### Rumen contents DNA extraction and archaea and bacterial Amplicon-Sequencing analysis

Total genomic DNA was isolated from rumen digesta using the repeated bead beating plus column (RBB + C) method, which is previous described by Yu et al.^103^. Briefly, the quality and quantity of DNA was measured using a NanoDrop Spectrophotometer ND-1000 (Thermo Fisher Scientific Inc., Wilmington, Delaware, USA).

The Amplicon-Seq analysis was performed using Quantitative Insights into Microbial Ecology 2, (QIIME2) (Ref.^104^; http://qiime.org/). Amplicon sequence libraries were processed, and low-quality ends of reads and primers were trimmed. The forward primer Arc915aF-CS1 ACACTGACGACATGGTTCTACAAGGAATTGGCGGGGGAGCAC and reverse primer Arc1386R-CS2 TACGGTAGCAGAGACTTGGTCTGCGGTGTGTGCAAGGAGC were used to amplify the archaea 16S rRNA gene amplicon region. The forward primer Bac9F-CS1 ACACTGACGACATGGTTCTACAGAGTTTGATCMTGGCTCAG and reverse primer Bac515R-CS2 TACGGTAGCAGAGACTTGGTCTCCGCGGCKGCTGGCAC were used to amplify the bacteria 16S rRNA gene region. The database used to align the archaea reads was Rumen and Intestinal Methanogen-DB (RIM-DB)^105^ and the database used to align the bacteria reads was Silva ribosomal RNA gene database Release 132^106–109^. A feature table and representative sequences table for bacteria and archaea profiles for each breed was generated for further analysis. Using these tables, a taxonomy file including ASV abundance for archaea and bacteria were generated. Detailed information regarding the animal management and condition during the rumen digesta collection is available in Li et al.^15^.

### Comparison of rumen archaea and bacteria taxonomic profiles between low and high-RFI groups across each pure breed and composite hybrid

Using QIIME2 command ‘qiime feature-table relative-frequency’, an output of the taxonomic ASV abundance based on total counts of each ASV in each taxa level (Kingdom, Phylum, Class, Order, Family, Genus, Species), for each animal was generated. Tables were then edited to have one table for each breed incorporating the ASV abundance for each taxonomic level, the taxonomic identification names, and the sample information. The ASV were analyzed regarding the differential abundance at genus level for bacteria and species level for archaea. Only taxonomic groups with an overall relative abundance > 0.5% were retained for the differential abundance analysis. Subsequently, for each animal within each breed, the relative abundance was calculated for the retained ASVs. To determine the significantly differentially abundant ASVs between high and low-RFI animals within each breed analysis (p-value < 0.05), the relative abundances were arcsine square root-transformed and a t-test was applied as described by Li et al.^15^.

### Identification of correlations between differentially abundant ASVs and gene expression

Initially, a sPLA-DA was performed for each breed individually using the read counts for those genes with |log_2_(FC)|> 2 in the comparison between high- and low-RFI groups. The sPLA-DA was performed using the mixOmics R package with the read counts standardized to zero means and unit variances^110^. The selectVar() function from mixOmics package was used to select a subset of genes which better differentiated the high and low-RFI groups based on the feature loadings from the first principal component of the sPLA-DA. The Spearman correlation between the read counts of the selected genes for each breed and the arcsine square root-transformed relative abundance of respective differentially abundant ASVs were estimated using the cor.test() function in R^111^. The significant correlations were defined as those with a p-value < 0.05. Subsequently, the ToppCluster software was used to perform a comparative clustering of metabolic pathways annotated for the genes significantly correlated with different ASVs within each breed^112^.

### Gene Ontology annotation and enrichment analysis

The R packages ClusterProfiler and enrichplot were used for gene ontology (GO) term enrichment analysis, graphic representation and functional grouping^113^. The GO enrichment analysis was performed for each breed for all the genes with |log_2_(FC)|> 2, for the subset of genes selected in the sPLS-DA analysis, and for each subset of genes correlated with each differentially abundant ASV. For GO terms annotated for all the genes with |log_2_(FC)|> 2, the function pairwise_termsim() from enrichplot was used to calculate the Jaccard correlation coefficient. The terms were functionally grouped using the similarity matrix built by the Jaccard correlation coefficient to identify terms functionally closely related and reduce redundancy across terms.

### Supplementary Information

Supplementary Figure 1.

Supplementary Figure 2.

Supplementary Table 1.

Supplementary Table 2.

Supplementary Table 3.

Supplementary Table 4.

Supplementary Table 5.

Supplementary Table 6.

Supplementary Table 7.

## Data Availability

The distribution of gene expression data can be accessed at https://www.cattleomics.com/transcriptome. The metagenome reads are available at National Center for Biotechnology Information (NCBI) under the accession ID PRJNA448333.
