# Supplementary material for: Multi-breed host rumen epithelium transcriptome and microbiome associations and their relationship with beef cattle feed efficiency
Source: Sci Rep. 2023 Sep 27;13:16209. doi: 10.1038/s41598-023-43097-8 (PMC10533831; doi:10.1038/s41598-023-43097-8)
Supplement: Supplementary file 1 — Supplementary Figure 1. [file 41598_2023_43097_MOESM1_ESM.pdf]

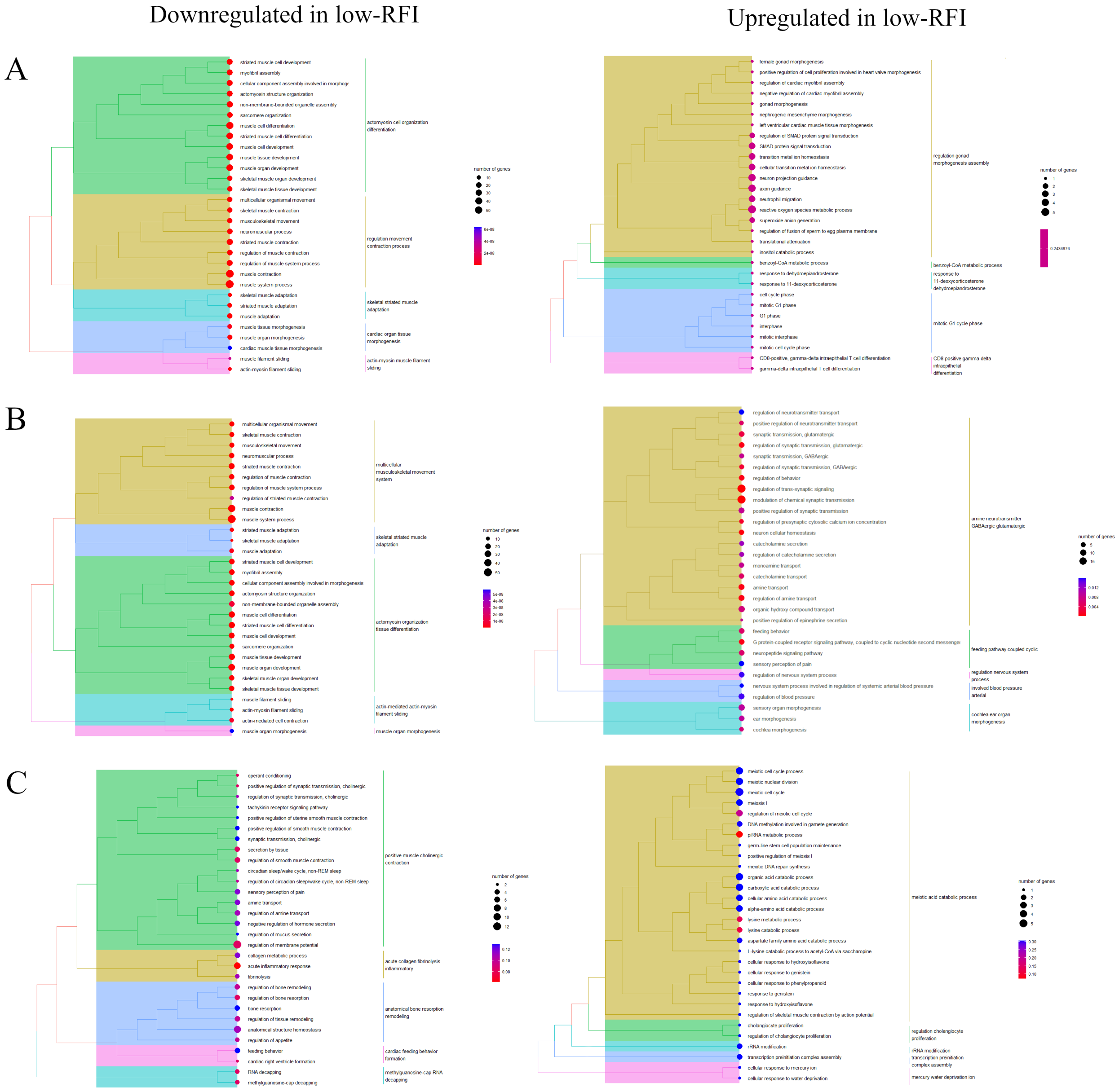

Supplementary Figure 1: Functional grouping of annotated gene ontology terms for up (right-hand side) and downregulated (left-hand side) genes in low-RFI groups for Angus (A), Kinsella (B) and Charolais (C) breeds.
