# Supplementary material for: Multi-breed host rumen epithelium transcriptome and microbiome associations and their relationship with beef cattle feed efficiency
Source: Sci Rep. 2023 Sep 27;13:16209. doi: 10.1038/s41598-023-43097-8 (PMC10533831; doi:10.1038/s41598-023-43097-8)
Supplement: Supplementary file 2 — Supplementary Figure 2. [file 41598_2023_43097_MOESM2_ESM.pdf]

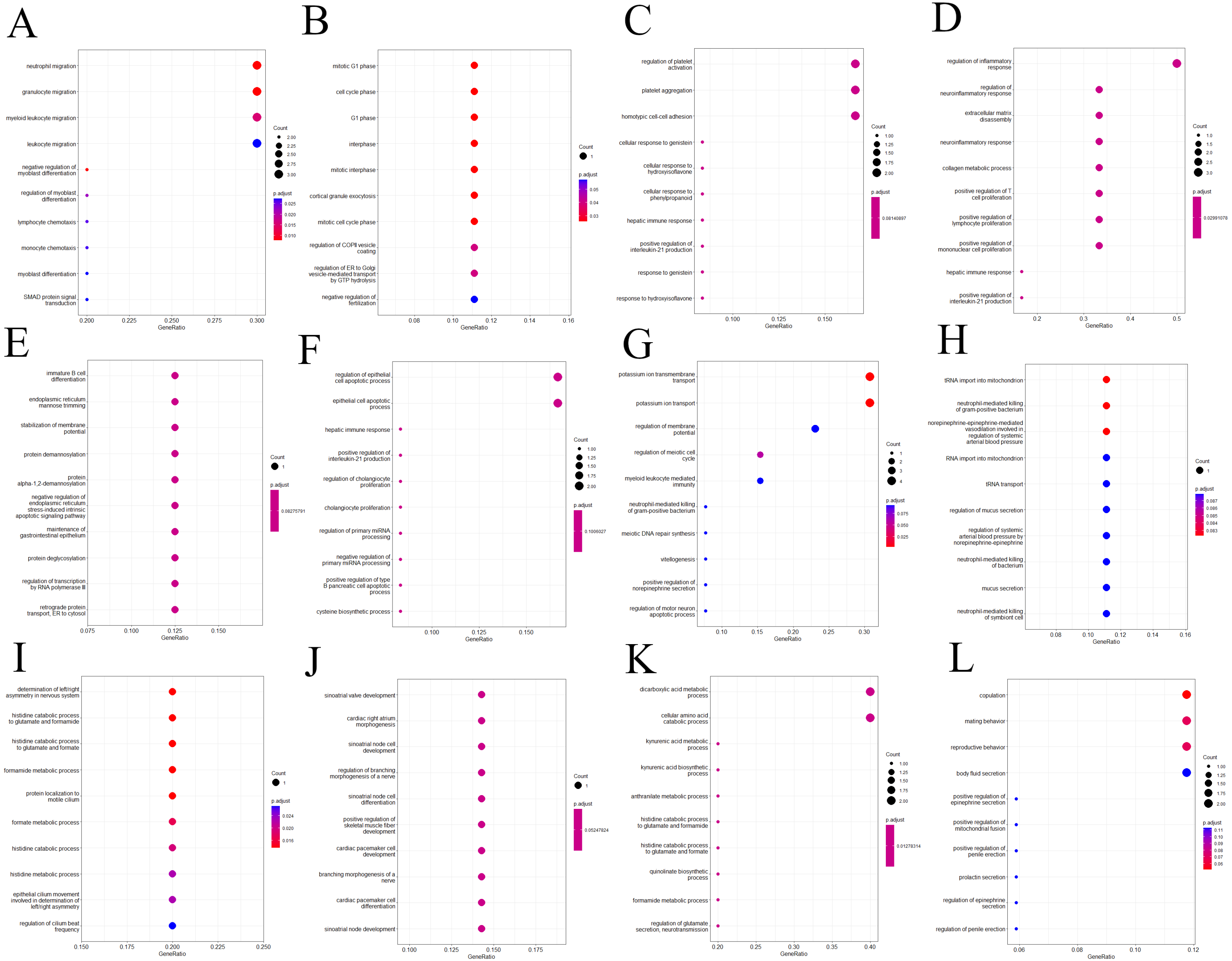


**Supplementary Figure 2:** Top 10 enriched gene ontology terms for the genes significantly correlated with *Ruminococcus 1* (A) and *Bacteroidales RF16 group* (B) in Angu *Erysipelotrichaceae UCG-004* (C), *Oribacterium* (D), *Prevotellaceae UCG-001* (E), *Clostridiales Family XIIID* (F), and *Clostridiales vadinBB60* (G) in Charolais; and *Christensenellaceae R-* *group* (H), *Ruminococcus 1* (I), *Papillibacter* (J), *Ruminococcus gauvreauii group* (K), and *Prevotellaceae UCG-001* (L) in Kinsella.
