# Supplementary material for: Multi-breed host rumen epithelium transcriptome and microbiome associations and their relationship with beef cattle feed efficiency
Source: Sci Rep. 2023 Sep 27;13:16209. doi: 10.1038/s41598-023-43097-8 (PMC10533831; doi:10.1038/s41598-023-43097-8)
Supplement: Supplementary file 3 — Supplementary Table 1. [file 41598_2023_43097_MOESM3_ESM.docx]

**Supplementary Table 1.** Descriptive alignment statistics for RNA-Sequence read alignment of low and high-RFI groups of samples in pure (Angus, Charolais) and composite hybrid (Kinsella) beef breeds.

| **Breed**  **RFI group** | **Animal ID** | **Total reads mapped** | **Uniquely mapped %** | **Non-specifically mapped %** | **Unmapped %** |
| --- | --- | --- | --- | --- | --- |
| Angus |  |  |  |  |  |
| Low-RFI | 301 | 38,759,070 | 91.60 | 6.17 | 2.22 |
|  | 302 | 42,946,978 | 91.05 | 6.73 | 2.22 |
|  | 307 | 31,711,494 | 91.56 | 6.42 | 2.02 |
|  | 401 | 37,452,936 | 89.79 | 6.80 | 3.41 |
|  | 407 | 34,318,440 | 56.32 | 4.23 | 39.44 |
|  | 501 | 33,489,466 | 89.26 | 7.27 | 3.48 |
|  | 505 | 40,185,986 | 91.79 | 6.44 | 1.77 |
|  | 506 | 44,563,794 | 92.08 | 6.56 | 1.36 |
| Average |  | 37,928,521 | 86.68 | 6.33 | 6.99 |
| High-RFI | 303 | 45,405,544 | 92.35 | 5.89 | 1.75 |
|  | 304 | 41,179,060 | 91.03 | 6.98 | 1.99 |
|  | 305 | 42,673,070 | 92.05 | 6.24 | 1.71 |
|  | 308 | 43,775,298 | 91.59 | 6.78 | 1.62 |
|  | 402 | 40,119,758 | 91.25 | 6.66 | 2.09 |
|  | 403 | 38,042,184 | 90.75 | 7.15 | 2.10 |
|  | 404 | 31,709,766 | 86.65 | 7.58 | 5.77 |
|  | 503 | 36,053,454 | 92.52 | 5.99 | 1.49 |
| Average |  | 39,869,767 | 91.02 | 6.66 | 2.32 |
| Charolais |  |  |  |  |  |
| Low-RFI | 604 | 51,048,594 | 91.46 | 7.09 | 1.45 |
|  | 611 | 40,709,670 | 91.18 | 6.68 | 2.14 |
|  | 612 | 44,199,494 | 91.22 | 7.11 | 1.67 |
|  | 702 | 34,201,080 | 91.90 | 6.67 | 1.43 |
|  | 704 | 40,308,908 | 90.50 | 7.36 | 2.13 |
|  | 706 | 35,073,386 | 90.33 | 7.69 | 1.98 |
|  | 710 | 39,102,138 | 91.19 | 6.97 | 1.84 |
|  | 712 | 33,133,416 | 92.01 | 6.60 | 1.39 |
| Average |  | 39,722,086 | 91.22 | 7.02 | 1.75 |
| High-RFI | 601 | 39,579,068 | 91.10 | 6.88 | 2.01 |
|  | 608 | 38,260,372 | 90.65 | 6.57 | 2.79 |
|  | 701 | 32,875,842 | 90.40 | 6.85 | 2.75 |
|  | 703 | 36,188,318 | 90.83 | 7.40 | 1.77 |
|  | 705 | 41,140,472 | 90.87 | 6.83 | 2.30 |
|  | 707 | 32,092,800 | 89.75 | 7.86 | 2.39 |
|  | 708 | 42,401,790 | 90.91 | 7.22 | 1.87 |
|  | 709 | 37,424,654 | 87.15 | 7.59 | 5.25 |
| Average |  | 37,495,415 | 90.22 | 7.15 | 2.64 |
| Kinsella |  |  |  |  |  |
| Low-RFI | 101 | 53,112,042 | 89.51 | 6.79 | 3.70 |
|  | 103 | 46,401,356 | 89.66 | 7.32 | 3.02 |
|  | 104 | 48,780,584 | 90.80 | 6.15 | 3.05 |
|  | 105 | 38,738,522 | 93.02 | 4.82 | 2.16 |
|  | 106 | 35,052,844 | 91.59 | 6.15 | 2.26 |
|  | 206 | 40,571,898 | 90.85 | 7.09 | 2.06 |
|  | 207 | 29,405,748 | 90.08 | 6.57 | 3.35 |
|  | 208 | 35,953,016 | 89.64 | 7.24 | 3.11 |
| Average |  | 41,001,001 | 90.64 | 6.52 | 2.84 |
| High-RFI | 107 | 40,604,336 | 90.81 | 6.80 | 2.40 |
|  | 112 | 41,161,658 | 88.90 | 7.91 | 3.18 |
|  | 201 | 41,666,324 | 90.18 | 7.43 | 2.38 |
|  | 202 | 47,721,354 | 90.93 | 6.56 | 2.51 |
|  | 203 | 38,334,790 | 90.67 | 6.44 | 2.89 |
|  | 205 | 38,399,802 | 90.50 | 6.74 | 2.76 |
|  | 209 | 38,816,174 | 91.15 | 6.42 | 2.43 |
|  | 210 | 45,303,556 | 92.08 | 6.01 | 1.91 |
| Average |  | 41,500,999 | 90.65 | 6.79 | 2.56 |
