# Supplementary material for: Multi-breed host rumen epithelium transcriptome and microbiome associations and their relationship with beef cattle feed efficiency
Source: Sci Rep. 2023 Sep 27;13:16209. doi: 10.1038/s41598-023-43097-8 (PMC10533831; doi:10.1038/s41598-023-43097-8)
Supplement: Supplementary file 4 — Supplementary Table 2. [file 41598_2023_43097_MOESM4_ESM.docx]

**Supplementary Table 2.** Descriptive alignment statistics for Amplicon-Sequence read alignment of low and high-RFI groups of samples in pure (Angus, Charolais) and composite hybrid (Kinsella) beef breeds.

| **Sample ID** | **Total # reads** | **Total # reads mapped** | **% reads mapped** |
| --- | --- | --- | --- |
| Angus |  |  |  |
| 301 | 63,628 | 51,014 | 80.18% |
| 302 | 39,710 | 32,566 | 82.01% |
| 303 | 56,360 | 44,277 | 78.56% |
| 304 | 61,416 | 49,432 | 80.49% |
| 305 | 61,236 | 50,606 | 82.64% |
| 307 | 56,384 | 45,409 | 80.54% |
| 308 | 56,770 | 48,086 | 84.70% |
| 401 | 75,808 | 62,417 | 82.34% |
| 402 | 57,154 | 44,976 | 78.69% |
| 403 | 52,272 | 41,622 | 79.63% |
| 404 | 67,368 | 53,964 | 80.10% |
| 407 | 60,930 | 49,254 | 80.84% |
| 501 | 57,220 | 47,328 | 82.71% |
| 503 | 59,614 | 48,490 | 81.34% |
| 505 | 54,164 | 45,162 | 83.38% |
| 506 | 76,516 | 61,967 | 80.99% |
| Average: | 59,784.38 | 48,535.63 | 81.20% |
| Total: | 956,550.00 | 776,570.00 |  |
|  |  |  |  |
| Charolais |  |  |  |
| 601 | 61,918 | 50,688 | 81.86% |
| 604 | 55,424 | 43,646 | 78.75% |
| 608 | 75,322 | 61,139 | 81.17% |
| 611 | 67,792 | 55,069 | 81.23% |
| 612 | 64,840 | 51,464 | 79.37% |
| 701 | 65,502 | 54,035 | 82.49% |
| 702 | 65,652 | 53,164 | 80.98% |
| 703 | 66,026 | 53,014 | 80.29% |
| 704 | 61,104 | 50,288 | 82.30% |
| 705 | 55,428 | 43,973 | 79.33% |
| 706 | 57,472 | 47,109 | 81.97% |
| 707 | 64,840 | 53,072 | 81.85% |
| 708 | 53,854 | 45,433 | 84.36% |
| 709 | 66,238 | 54,658 | 82.52% |
| 710 | 63,780 | 52,010 | 81.55% |
| 712 | 72,168 | 57,405 | 79.54% |
| Average: | 63,585.00 | 51,635.44 | 81.22% |
| Total: | 1,017,360.00 | 826,167.00 |  |
| Kinsella |  |  |  |
| 101 | 55,784 | 44,293 | 79.40% |
| 103 | 60,500 | 49,294 | 81.48% |
| 104 | 66,080 | 53,430 | 80.86% |
| 105 | 48,644 | 39,568 | 81.34% |
| 106 | 58,792 | 47,885 | 81.45% |
| 107 | 48,356 | 38,933 | 80.51% |
| 112 | 61,318 | 51,060 | 83.27% |
| 201 | 78,004 | 62,805 | 80.52% |
| 202 | 51,368 | 40,685 | 79.20% |
| 203 | 58,090 | 46,986 | 80.88% |
| 205 | 53,278 | 42,446 | 79.67% |
| 206 | 57,414 | 46,585 | 81.14% |
| 207 | 63,820 | 51,107 | 80.08% |
| 208 | 50,404 | 42,311 | 83.94% |
| 209 | 71,864 | 58,053 | 80.78% |
| 210 | 73,590 | 59,172 | 80.41% |
| Average: | 59,831.63 | 48,413.31 | 80.93% |
| Total: | 957,306.00 | 77,4613.00 |  |
